# Does Further Lowering Intraoperative Intraocular Pressure Reduce Surgical Invasiveness in Active-Fluidics Eight-Chop Phacoemulsification? A Fellow-Eye Comparative Study

**DOI:** 10.3390/jcm15010366

**Published:** 2026-01-04

**Authors:** Tsuyoshi Sato

**Affiliations:** Department of Ophthalmology, Sato Eye Clinic, Nemoto 3-3, Matsudo-shi 271-0077, Chiba-ken, Japan; perfect-eightchop@sato-ganka.com

**Keywords:** aqueous flare, cataract surgery, corneal endothelial cell, eight-chop technique, phacoemulsification, low intraocular pressure setting

## Abstract

**Background**: Active-fluidics phacoemulsification can maintain anterior chamber stability at lower intraoperative intraocular pressure (IOP) levels. However, whether reducing IOP alone—without additional stabilizing technologies such as the Active Sentry handpiece—can decrease surgical invasiveness during Eight-Chop phacoemulsification remains unclear. **Methods:** In this prospective fellow-eye comparative study, 56 non-diabetic patients (112 eyes) underwent Eight-Chop technique phacoemulsification using the Centurion Vision System with active fluidics. One eye was randomly assigned to a standard-IOP setting (55 mmHg; high-IOP group) and the fellow eye to a reduced-IOP setting (28 mmHg; low-IOP group). Intraoperative parameters—including operative time, phaco time, aspiration time, cumulative dissipated energy (CDE), and irrigation volume—were recorded. Postoperative outcomes included aqueous flare (laser flare photometry), corneal endothelial cell density (CECD) and CECD loss, corneal morphology (central corneal thickness [CCT], coefficient of variation [CV], percentage of hexagonal cells [PHC]), and IOP. Linear mixed-effects models with patient ID as a random effect were used for all paired-eye comparisons. **Results:** Lowering the intraoperative IOP did not reduce surgical invasiveness. Phaco time was significantly longer in the low-IOP group (16.2 ± 5.22 s vs. 13.9 ± 4.40 s; *p* = 0.001), and aspiration time was also longer (75.0 ± 18.3 s vs. 69.0 ± 17.9 s; *p* = 0.033). No significant differences were found in operative time (5.08 ± 1.10 min vs. 4.82 ± 1.13 min; *p* = 0.082), CDE (5.93 ± 1.87 vs. 5.56 ± 1.90; *p* = 0.099), or irrigation volume (26.6 ± 7.71 mL vs. 25.2 ± 7.35 mL; *p* = 0.214). Postoperative outcomes were similarly comparable. Aqueous flare showed no significant differences at any time point (e.g., day 1: 14.8 ± 5.10 vs. 14.5 ± 4.76 ph/ms; *p* = 0.655). Mean CECD loss remained small in both groups and did not differ significantly (7 weeks: −0.82 ± 1.05% vs. −0.98 ± 1.16%, *p* = 0.460; 19 weeks: −0.93 ± 1.38% vs. −1.28 ± 1.69%, *p* = 0.239). Corneal morphological parameters (CCT, CV, PHC) and postoperative IOP also showed no significant differences between settings. **Conclusions:** In this fellow-eye comparative study, lowering intraoperative intraocular pressure from conventional to near-physiologic levels under active-fluidics control did not reduce surgical invasiveness during Eight-chop phacoemulsification. No additional benefits were observed in terms of endothelial cell preservation, postoperative inflammation, or overall surgical performance. These findings indicate that, when chamber stability is already ensured by a low-invasive fragmentation strategy, further reduction in intraoperative IOP alone does not confer measurable short-term clinical advantages.

## 1. Introduction

Since its introduction in 1967, phacoemulsification has become the global standard for cataract surgery [1]. Although continuous technological innovations have greatly enhanced the safety and efficiency of the procedure, maintaining intraoperative anterior chamber stability remains a critical challenge. Sudden fluctuations in intraocular pressure (IOP) can induce fluidic surge, causing capsular rupture or endothelial injury due to transient pressure spikes. Technologies that preserve chamber stability at appropriate IOP levels are therefore essential for safe, minimally invasive surgery [2].

Traditionally, gravity-based fluidics systems (GFS) regulate irrigation flow by adjusting bottle height, but this method cannot compensate for aspiration fluctuations, often resulting in chamber instability and IOP spikes [3]. The Active Fluidics System (AFS) was developed to dynamically modulate irrigation pressure using real-time sensors, improving chamber stability even at lower IOP settings [4]. Several studies have shown reduced corneal edema and endothelial cell loss with AFS compared with GFS [5,6,7]. However, most previous reports incorporated the Active Sentry handpiece, making it difficult to isolate the intrinsic performance of AFS alone. From a mechanistic standpoint, lowering intraoperative IOP has been hypothesized to reduce mechanical stress on the corneal endothelium and intraocular tissues. However, in an active-fluidics system capable of dynamically maintaining chamber stability, the relative contribution of IOP re-duction alone remains unclear.

The eight-chop technique is a nucleus pre-segmentation method that may enhance surgical efficiency and reduce intraocular trauma [8]. Combining eight-chop with a pure AFS environment—specifically without Active Sentry assistance—provides an opportunity to directly evaluate the independent effects of different IOP settings on intraoperative performance and postoperative endothelial protection. In this study, the OZil ultrasound handpiece was used to maintain identical handpiece configurations across IOP conditions, enabling a precise assessment of IOP-dependent fluidic behavior under pure AFS control.

## 2. Materials and Methods

### 2.1. Ethical Considerations

This study adhered to the tenets of the Declaration of Helsinki and was conducted in accordance with all applicable institutional and national ethical regulations. The research protocol, including the paired-eye study design comparing two intraoperative IOP settings under an AFS, was reviewed and approved by the Institutional Ethics Committee of Sato Eye Clinic (approval number: 20250301). Prior to surgery, all patients received a detailed explanation regarding the purpose of the study and the use of their clinical data for research and publication. Written informed consent was obtained from every participant. No additional interventions outside standard clinical care were performed, and all procedures were carried out using established surgical techniques by a single experienced surgeon.

### 2.2. Study Population

This prospective paired-eye study enrolled 56 non-diabetic patients (112 eyes) who underwent routine cataract surgery using the Eight-chop technique at Sato Eye Clinic (Matsudo City, Chiba, Japan) between April 2025 and September 2025. To eliminate potential systemic influences on postoperative inflammation and corneal endothelial responses, only patients without diabetes mellitus were included. Randomization was performed at the eye level using a computer-generated random sequence to assign each eye to either the conventional-IOP or low-IOP setting. Allocation was concealed until the time of surgery by using a sealed assignment list prepared in advance. Because a fellow-eye design was employed, each patient served as their own control, thereby minimizing inter-individual variability. The randomization process was implemented solely to determine the IOP setting for each eye and did not affect any other surgical parameters. Each participant contributed both eyes, with one eye randomly assigned to undergo surgery under a standard intraoperative IOP setting (55 mmHg) and the fellow eye operated on under a reduced IOP setting (28 mmHg) using the same AFS. Eyes were excluded if any of the following conditions were present: corneal pathology or opacity; history of uveitis or glaucoma; retinal disease, including diabetic retinopathy; congenital anterior segment anomalies such as microcornea, nanophthalmos, or anterior segment dysgenesis; previous ocular trauma or intraocular surgery; lens nucleus Emery-Little Grade IV or higher [9]; the need for adjunctive surgical devices such as iris retractors or capsular tension rings; intraoperative complications; or inability to complete postoperative follow-up through 19 weeks. Because both eyes from each patient were included, intra-subject correlation was addressed analytically using a linear mixed-effects model. The order of surgery between fellow eyes was alternated across participants. In approximately half of the cases, the right eye was operated on first, and in the remaining cases, the left eye was operated on first. The assignment of intraoperative IOP settings was independent of surgical order. This approach was adopted to minimize potential learning effects or surgeon fatigue bias associated with sequential bilateral surgery.

### 2.3. Preoperative Assessment

Before surgery, all participants underwent a standardized preoperative ophthalmic evaluation. Best-corrected visual acuity (BCVA) was measured using a decimal chart and converted to logarithm of the minimum angle of resolution (logMAR) values for analysis. IOP was assessed with a non-contact tonometer under consistent measurement conditions. Corneal endothelial parameters—including corneal endothelial cell density (CECD), central corneal thickness (CCT), coefficient of variation in cell area (CV), and the percentage of hexagonal cells (PHC)—were recorded using a non-contact specular microscope (EM-3000, Topcon Corporation, Tokyo, Japan). Anterior chamber depth and axial length were measured with a swept-source optical biometer operating at a 1060 nm wavelength (OA-2000, TOMEY Corporation, Tokyo, Japan). All examinations were performed by trained technicians following uniform protocols to ensure measurement reliability across both IOP settings.

### 2.4. Active Fluidics System and Intraoperative IOP Settings

All surgeries were performed using an active fluidics phacoemulsification platform, which maintains target irrigation pressure by continuously adjusting peristaltic pump activity in response to real-time changes in aspiration flow. This system provides stable anterior chamber conditions during phacoemulsification without reliance on elevated bottle height. Two intraoperative IOP settings were defined for comparison in the present study. The standard setting (high-IOP group) used a target IOP of 55 mmHg, consistent with commonly adopted pressure levels intended to optimize chamber stability in conventional active fluidics cataract surgery. In contrast, the low-IOP group was operated under a reduced target IOP of 28 mmHg, approximating physiological pressure levels while still enabling the AFS to maintain chamber depth during aspiration and phacoemulsification. The low-IOP setting of 28 mmHg was selected because it lies within the clinically established low-pressure range (20–30 mmHg) reported in previous active-fluidics phacoemulsification studies [5,10]. The target IOP was set prior to surgery and remained constant throughout each procedure. No intraoperative adjustments were made. Importantly, no pressure-sensing handpiece technologies (such as Active Sentry) were employed, ensuring that the only intraoperative variable between paired eyes was the programmed IOP setting. This design allowed the study to directly evaluate whether lowering the intraoperative IOP, independent of additional stabilizing technologies, confers measurable benefits in terms of surgical invasiveness or postoperative tissue response.

### 2.5. Surgical Technique

All procedures were performed by a single experienced cataract surgeon (T.S.), who routinely conducts phacoemulsification using the Eight-chop technique. A 3.0 mm clear corneal incision was created with a steel keratome, and the anterior chamber was filled with a combination of cohesive and dispersive ophthalmic viscosurgical devices. A continuous curvilinear capsulorrhexis measuring approximately 6.0–6.2 mm was completed using capsulorrhexis forceps, followed by gentle hydrodissection with a 27-gauge cannula to mobilize the lens. Nuclear disassembly was performed using the Eight-chop technique. The nucleus was mechanically divided into eight small, uniformly sized fragments using the Eight-chopper II, without the use of any secondary instrument. Following fragmentation, phacoemulsification and aspiration of the divided nuclear pieces were performed in standard coaxial mode. All phacoemulsification and aspiration procedures were performed with the OZil ultrasound handpiece integrated into the Centurion^®^ Vision System (Alcon Laboratories, Fort Worth, TX, USA) operating under the active-fluidics platform. Aside from the programmed IOP setting (55 mmHg vs. 28 mmHg), all machine parameters were kept constant between paired eyes. Cortical material was removed using coaxial irrigation/aspiration. A foldable hydrophobic acrylic intraocular lens (AcrySof^®^ MN60AC; Alcon Laboratories, Geneva, Switzerland) was implanted into the capsular bag. Residual ophthalmic viscosurgical device was completely removed, and the anterior chamber was reformed with balanced salt solution containing moxifloxacin (0.5 mg/mL). The following intraoperative parameters were automatically recorded by the phacoemulsification system: phaco time, aspiration time, cumulative dissipated energy (CDE), and irrigation fluid usage. Operative time was measured manually from creation of the primary incision to the completion of viscoelastic removal. All surgeries were recorded using a high-definition digital surgical imaging system and archived for review.

### 2.6. Outcome Measures

Postoperative outcomes were evaluated to determine whether lowering the programmed intraoperative IOP provided measurable reductions in surgical invasiveness when phacoemulsification was performed using the Eight-chop technique under active fluidics control. All parameters were assessed at baseline and postoperatively at 1 day, 1 week, 7 weeks, and 19 weeks unless otherwise specified.

#### 2.6.1. Aqueous Flare

Aqueous flare, used as an indicator of postoperative anterior chamber inflammation, was measured with a laser flare photometer (FM-600; Kowa Co., Ltd., Nagoya, Japan). Three consecutive measurements were obtained at each visit, and the mean value (photon counts/ms) was used for analysis. Flare was evaluated at preoperative baseline and at postoperative day 1, week 1, week 7, and week 19.

#### 2.6.2. Corneal Endothelial Cell Density

Corneal endothelial cell density was measured using a non-contact specular microscope (EM-3000; Topcon Corporation, Tokyo, Japan). Automated cell counting was verified manually to ensure accuracy. CECD loss (%) was calculated as

CECD loss = (preoperative CECD − postoperative CECD)/preoperative CECD × 100

In accordance with previous reports demonstrating that increases in endothelial cell density fall within the test–retest variability of specular microscopy and are therefore biologically implausible, postoperative CECD increases were treated as 0% loss [11].

#### 2.6.3. Corneal Morphological Parameters

Morphological indices of the corneal endothelium—including CV, PHC, and CCT—were also measured with the same specular microscope. These variables provided complementary markers of endothelial stress and postoperative recovery. CCT, CV, and PHC were evaluated preoperatively and at postoperative weeks 7 and 19.

#### 2.6.4. Intraocular Pressure

IOP was measured with a non-contact tonometer (CT-1P; Topcon Corporation). IOP was assessed preoperatively and at weeks 7 and 19 postoperatively. Percentage change in IOP from baseline was also calculated to describe the magnitude of the postoperative IOP-lowering effect associated with cataract surgery.

#### 2.6.5. Best-Corrected Visual Acuity

BCVA was assessed using a decimal visual acuity chart and converted to logarithm of the minimum angle of resolution (logMAR) for analysis. Visual acuity was measured preoperatively and at postoperative weeks 7 and 19.

#### 2.6.6. Intraoperative Parameters

The Centurion^®^ Vision System automatically recorded ultrasound time, aspiration time, CDE, and total irrigation volume during surgery. Operative time, defined as the interval from the initial corneal incision to complete removal of the ophthalmic viscosurgical device, was manually recorded. These metrics were analyzed to determine whether lowering the intraoperative IOP influenced surgical efficiency.

### 2.7. Sample Size Considerations

Sample size was calculated for the primary outcome of the percentage change in CECD. Because each patient contributed one eye to each intraoperative IOP setting, a paired-eye design was assumed. Based on our previous Eight-chop cohort and published test–retest variability data for non-contact specular microscopy, we estimated the standard deviation of the paired difference in CECD loss to be approximately 2.5%. A 1.0% difference in CECD loss between settings was considered clinically relevant. Under these assumptions, a paired t-test effect size of dz = 0.40 (moderate effect) was used for power estimation. With a two-sided α of 0.05 and a power of 80%, a minimum of 49 paired eyes (49 patients) was required. In the present study, 56 patients were enrolled, exceeding the calculated requirement. Therefore, the sample size was considered sufficient to detect clinically meaningful differences in CECD loss between high- and low-IOP settings, despite partial missing values at postoperative follow-up visits.

### 2.8. Statistical Analysis

All statistical analyses were conducted using R software (version 4.5.0; R Foundation for Statistical Computing, Vienna, Austria) and RStudio (version 2025.09.2; Posit, PBC, Boston, MA, USA). Because each patient contributed one eye to the high-IOP setting and the fellow eye to the low-IOP setting, intra-patient correlation was accounted for using a linear mixed-effects models. Anonymized datasets supporting the analyses are provided as Supplementary Dataset 1 and Supplementary Dataset 2.

#### 2.8.1. Modeling Approach

For all continuous outcomes—including intraoperative parameters, postoperative aqueous flare, CECD loss, corneal morphological indices (CCT, CV, PHC), IOP, and best-corrected visual acuity (BCVA)—linear mixed-effects models were fitted with the following structure:

Outcome ∼ IOP setting (fixed effect) + (1∣patient ID)

The lmerTest package (version 3.1-3; Kuznetsova, Brockhoff and Christensen, R Foundation for Statistical Computing, Vienna, Austria) was used to compute *p*-values using Satterthwaite’s method. Model diagnostics included visual inspection of residual distribution and homogeneity of variance.

#### 2.8.2. Handling of Missing Data

Unequal numbers of observations at 7 and 19 weeks, mainly due to missed follow-up visits, were handled within the linear mixed-effects model framework, which accommodates unbalanced repeated-measures data under the missing-at-random assumption. No imputation was performed.

#### 2.8.3. Analysis of Corneal Endothelial Cell Density Loss

CECD loss (%) at weeks 7 and 19 was analyzed using a linear mixed-effects models with IOP setting as a fixed effect and patient ID as a random effect. Values representing postoperative increases in CECD were included as 0% loss in the models, consistent with the analytical policy specified in Section 2.6.2. Group differences at each postoperative time point were evaluated using two-tailed *p*-values.

#### 2.8.4. Analysis of Preoperative Characteristics

Age and sex were not statistically compared due to the paired-eye design producing identical distributions between groups. Other baseline ocular parameters were compared using the same linear mixed-effects model structure as described above.

#### 2.8.5. Visual Acuity Analysis

Decimal BCVA was converted to logMAR prior to analysis. Linear mixed-effects models were used to compare BCVA between high- and low-IOP settings at preoperative, week 7, and week 19 visits.

#### 2.8.6. Significance Threshold

All statistical tests were two-sided. A *p*-value < 0.05 was considered statistically significant. As the purpose of this study was to determine whether lowering intraoperative IOP improves postoperative outcomes relative to the standard setting, no adjustments for multiple comparisons were applied, which aligns with previous paired-eye analytical approaches in cataract surgery research.

### 2.9. GenAI Statement

During the preparation of this manuscript, the author used ChatGPT (GPT-5 mini; OpenAI, San Francisco, CA, USA) solely to assist with English language editing and to improve clarity and readability. All scientific content, data interpretation, statistical analyses, and conclusions were entirely conducted and verified by the author.

## 3. Results

A total of 112 eyes from 56 non-diabetic patients were included in the analysis, with one eye assigned to surgery under the standard intraoperative IOP setting (high-IOP group) and the fellow eye operated on under a reduced IOP setting (low-IOP group) using an AFS. All procedures were completed using the Eight-chop phacoemulsification technique by a single experienced surgeon.

### 3.1. Preoperative Characteristics

Preoperative demographic and ocular characteristics were well balanced between the two IOP settings (Table 1). There were no relevant differences between groups in age, sex distribution, axial length, anterior chamber depth, or lens nucleus grade. Similarly, baseline corneal parameters including CCT, CV, PHC, and CECD were comparable between the high- and low-IOP eyes. Preoperative IOP and aqueous flare values also did not differ significantly between settings, indicating that the paired-eye design yielded two groups with similar starting conditions.

### 3.2. Intraoperative Parameters

Intraoperative surgical parameters are summarized in Table 2. The overall operative time did not differ significantly between the high- and low-IOP settings; mean surgery duration was approximately 5 min in both groups, and the linear mixed-effects model did not show a significant effect of IOP setting on operative time. CDE and the volume of balanced salt solution (BSS) used were likewise similar between groups, with no statistically significant differences. In contrast, both ultrasound time and aspiration time tended to be longer in the low-IOP group. Mixed-effects modeling showed that ultrasound time was significantly increased under low-IOP conditions compared with the standard setting, and aspiration time was also modestly but significantly prolonged in the low-IOP group. These findings suggest that lowering intraoperative IOP in this AFS did not enhance surgical efficiency and, in this cohort, was associated with somewhat longer phaco and aspiration times.

### 3.3. Postoperative Flare and Corneal Endothelial Outcomes

**Figure 1:**
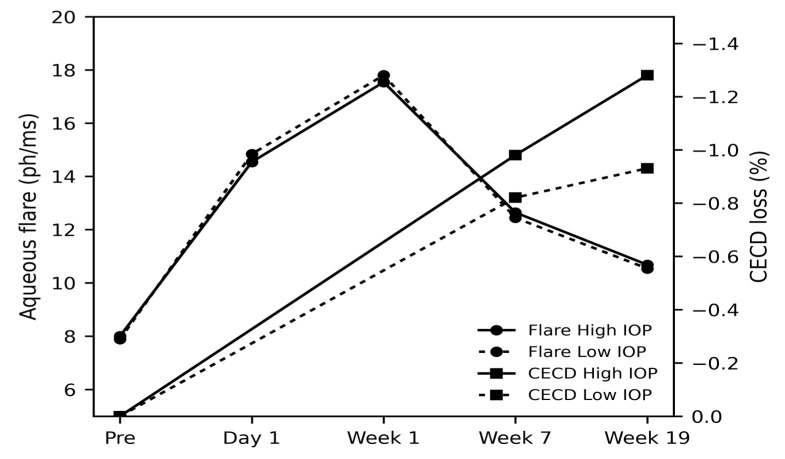
Temporal changes in aqueous flare and CECD loss after surgery in high- and low-IOP settings. Aqueous flare values are shown on the left *y*-axis, and CECD loss percentages on the right *y*-axis. CECD loss was defined as 0% at baseline (Pre). Values represent group means.

Postoperative aqueous flare showed the typical temporal pattern after uneventful phacoemulsification in both groups, with a marked increase on postoperative day 1 followed by gradual decline over the subsequent weeks (Table 3 and Figure 1). However, at none of the examined time points—preoperatively, 1 day, 1 week, 7 weeks, or 19 weeks—did the low-IOP setting demonstrate a reduction in flare compared with the high-IOP setting. Linear mixed-effects models with a random intercept for patient demonstrated no significant effect of IOP setting on flare at any time point, with all *p*-values well above 0.05. Thus, reducing intraoperative IOP to a near-physiological level did not translate into a measurable decrease in early postoperative anterior chamber inflammation.

Corneal endothelial cell density outcomes were favorable overall, reflecting the low-invasiveness of the Eight-chop technique, but no clear advantage of the low-IOP setting was observed (Table 3 and Figure 1). Mean endothelial cell loss at 7 weeks was approximately 1% in both groups, and at 19 weeks remained on the order of 1–1.5% in each group, when increases in CECD were treated as 0% loss. Mixed-effects analysis showed no significant difference in percentage CECD loss between the high- and low-IOP settings at either 7 or 19 weeks, with *p*-values of 0.46 and 0.24, respectively. In other words, while endothelial loss remained remarkably small in this series, lowering the intraoperative IOP did not further reduce CECD loss beyond that achieved with the standard IOP setting.

### 3.4. Corneal Morphological Parameters

Corneal morphological parameters followed a similar pattern. CCT exhibited minimal changes from baseline to 7 and 19 weeks in both groups, and there were no significant differences in CCT between the high- and low-IOP eyes at any time point (Table 4). Likewise, CV and PHC remained within physiologic ranges throughout follow-up, and group comparisons using a linear mixed-effects models revealed no significant impact of IOP setting on these indices of endothelial cell morphology. Taken together, these results indicate that, within the present sample, reducing intraoperative IOP did not produce discernible improvements in postoperative corneal structural metrics compared with the standard setting.

### 3.5. Postoperative Intraocular Pressure

Postoperative IOP also remained stable and similar between groups (Table 5). Both high- and low-IOP eyes exhibited a modest reduction in IOP from preoperative values to 7 and 19 weeks postoperatively, consistent with the expected IOP-lowering effect of uncomplicated cataract surgery. However, no significant differences in IOP were observed between the two IOP settings at any postoperative time point in the mixed-effects analysis. Therefore, lowering intraoperative IOP during surgery did not influence the longer-term IOP profile in this cohort.

### 3.6. Best-Corrected Visual Acuity

Best-corrected visual acuity (BCVA) improved substantially after surgery in both groups (Table 6). Preoperatively, mean logMAR BCVA was slightly above 0.10 in each group, reflecting mild to moderate visual impairment from cataract. By 7 weeks postoperatively, mean BCVA improved to approximately −0.07 logMAR in both the high- and low-IOP eyes, and remained stable through 19 weeks. Mixed-effects models showed no significant differences in BCVA between the two IOP settings at baseline, 7 weeks, or 19 weeks (all *p* > 0.33). Thus, lowering intraoperative IOP did not confer additional visual benefit beyond that obtained with standard-IOP Eight-chop phacoemulsification.

### 3.7. Intraoperative Stability at Low IOP

In this study, the Active Sentry handpiece (Alcon Laboratories, Fort Worth, TX, USA) was not used; therefore, quantitative evaluation of Active Surge Mitigation was not possible. Although the anterior chamber appeared slightly less stable at the lower IOP setting compared with the higher setting, no clinically relevant surge events occurred, and no intraoperative complications were observed in any eye.

### 3.8. Complications

No intraoperative or postoperative complications occurred in either group throughout the study period. All procedures were completed without posterior capsule rupture, zonular dehiscence, thermal injury, or the need for unplanned surgical maneuvers such as conversion to an alternative technique or use of capsular tension devices. Postoperatively, no eye exhibited persistent corneal edema, wound leakage, IOP spikes requiring treatment, or inflammatory reactions beyond the expected transient response after phacoemulsification. Across the entire cohort, the postoperative course was stable and uneventful, and no complication could be attributed to the intraoperative IOP setting in either surgical condition.

## 4. Discussion

The present fellow-eye comparative investigation was designed to clarify whether lowering intraoperative IOP under an active-fluidics environment confers measurable reductions in surgical invasiveness during Eight-chop phacoemulsification. A major advancement in modern fluidics technology has been the transition from traditional GFS to AFS [6,12,13,14]. Whereas GFS relies on high bottle heights of approximately 70–110 cm to maintain adequate inflow, this approach inevitably increases IOP and predisposes the eye to sudden pressure fluctuations during changes in vacuum. Such pressure elevations and instability have been associated with adverse events, including subtle retinal ischemia, endothelial cell loss, postoperative inflammation, and a heightened risk of posterior capsule rupture [15,16]. In contrast, AFS maintains anterior chamber stability through real-time pressure modulation, minimizing IOP surges and reducing the need for excessively high irrigation pressure [6,12,13,14]. Previous reports have also suggested that lower infusion pressure may be more favorable for corneal endothelial preservation [15,16]. These fluidic advantages provide a strong rationale for investigating whether further lowering the target IOP within an AFS environment can enhance the minimally invasive nature of phacoemulsification. The Eight-chop technique is characterized by complete mechanical segmentation of the lens nucleus into eight uniformly sized fragments before ultrasonic emulsification [8]. By establishing full-thickness cleavage planes in advance, Eight-chop minimizes the need for high phaco power, improves aspiration efficiency, and reduces total irrigation volume by enabling faster fragment removal—factors known to influence endothelial safety and postoperative inflammation [17,18,19]. Modern AFSs allow surgeons to select a wide range of target IOP settings while maintaining chamber stability through real-time pressure modulation [6,10,12,20]. However, despite this technological flexibility, it has remained uncertain whether decreasing the target IOP below conventional levels provides additional advantages with respect to intraoperative tissue stress or early postoperative inflammatory response, particularly when combined with a fragmentation strategy that is inherently low-invasive such as Eight-chop. To address this unresolved issue, the present study isolated the active-fluidics IOP setting as a single independent variable while keeping all other surgical parameters—including the surgeon, instrumentation, phaco power modulation, and fragmentation method—strictly constant. This design enabled a direct evaluation of whether further lowering intraoperative IOP yields incremental benefits in reducing surgical invasiveness. It is important to emphasize that the present study was not designed to compare the Eight-chop technique with other nucleus fragmentation methods, nor to establish its superiority over alternative surgical strategies or fluidic configurations. Rather, the objective of this investigation was narrowly focused on determining whether lowering intraoperative IOP alone provides additional reductions in surgical invasiveness when all other surgical variables are held constant within an Eight-chop–based framework. Accordingly, the absence of measurable benefits from IOP reduction should be interpreted as evidence that, under the specific conditions of this study, further modulation of IOP does not yield incremental advantages beyond those already conferred by the established surgical strategy, rather than as proof of the inherent dominance of the Eight-chop technique itself. From a mechanistic standpoint, lowering intraoperative IOP has been hypothesized to reduce mechanical stress on the corneal endothelium and intraocular tissues. However, in an active-fluidics system capable of dynamically maintaining chamber stability, the relative contribution of IOP reduction alone remains unclear. Our findings suggest that, when chamber stability is already well preserved by the intrinsic efficiency of the Eight-chop technique, further IOP reduction without additional stabilizing feedback does not confer measurable reductions in surgical invasiveness.

Because the Active Sentry handpiece was not used in the present study, direct assessment of Active Surge Mitigation [12,13] could not be performed. Nevertheless, despite the lower target IOP, no clinically significant surge or aspiration-related chamber collapse occurred. Although the anterior chamber exhibited slightly reduced stability at the lower IOP setting, this did not affect surgical safety or phacoemulsification performance. Several factors intrinsic to the Eight-chop technique likely contributed to this favorable outcome. First, Eight-chop achieves complete mechanical segmentation of the nucleus before ultrasound emulsification, allowing highly efficient removal of each small fragment with minimal phaco power and rapid aspiration, thereby reducing the overall irrigation requirement. Second, the procedure does not require creation of a side port for a second instrument, thereby eliminating leakage of irrigation fluid through the paracentesis—a common source of chamber instability at low IOP. Third, because no second instrument is inserted, there is no external compression or distortion of the globe, which prevents deformation of the main incision and further reduces outflow leakage. Collectively, these characteristics help maintain inflow–outflow balance and promote stable intraoperative fluidics even at reduced IOP. Thus, although chamber stability is inherently more vulnerable at low pressure, the structural features of Eight-chop minimized the likelihood of surge and preserved overall surgical safety.

An essential methodological element in the design of this study was the deliberate omission of the Active Sentry handpiece. Previous investigations examining low-IOP surgery have combined this modality with Active Sentry technology [7,12,13], which incorporates a pressure-sensing mechanism capable of providing real-time intraocular compensation independent of the preset IOP target. From a scientific standpoint, introducing Active Sentry handpiece would add a second stabilizing variable and complicate the interpretation of postoperative outcomes. Any improvements observed under such dual-stabilized configurations could arise from the lower IOP, from the autonomous compensatory action of the handpiece, or from an interaction between the two factors. To avoid this source of confounding and maintain strict internal validity, the present study standardized all surgeries to a single active-fluidics platform and modified only the intraoperative IOP target. This approach enabled a pure evaluation of whether reducing IOP to a near-physiologic level has an independent and clinically meaningful impact on postoperative inflammation, endothelial preservation, or surgical efficiency within the Eight-chop-based surgical strategy.

The chosen IOP values of 55 mmHg for the conventional setting and 28 mmHg for the reduced setting were selected based on previously reported ranges considered representative of standard- and low-pressure phacoemulsification under active fluidics. Numerous studies have situated low-IOP surgical conditions between approximately 20 and 30 mmHg, while conventional values commonly range from 50 to 60 mmHg [7,21,22]. By selecting pressures that align closely with these established ranges, the present study ensured the use of clinically relevant parameters that facilitate appropriate comparison with the broader cataract surgery literature. The study design therefore enables conclusions that extend beyond an isolated surgical context and are compatible with modern fluidics standards.

The primary finding of this study is that reducing intraoperative IOP to 28 mmHg did not produce meaningful improvements in intraoperative performance metrics compared with the standard setting. Although operative time, cumulative dissipated energy, and irrigation volume were similar between groups, both phaco time and aspiration time were significantly prolonged under low-IOP conditions. This suggests that reduced IOP may negatively affect followability and vacuum-related fragment engagement, even when the AFS attempts to compensate for chamber fluctuations. In Eight-chop, the nucleus is pre-divided into small fragments that are readily aspirated. However, the findings of this study suggest that when intraoperative IOP is lowered below a certain threshold, the efficiency with which fragments move toward the phaco tip may be hindered, thereby diminishing the intrinsic fluidic advantages of the Eight-chop technique.

Aqueous flare behavior provides additional insight into the biological implications of IOP manipulation. Flare measurements reflect early postoperative breakdown of the blood–aqueous barrier and are therefore sensitive indicators of intraocular inflammation. In the present study, flare values remained consistently low throughout the postoperative period in both IOP groups, with no significant differences at any time point. This pattern strongly suggests that the Eight-chop technique itself exerts a dominant influence over postoperative inflammatory response, minimizing factors such as uveal irritation and thermal exposure. Because the nucleus is fully segmented mechanically before ultrasound is applied, intraocular stress is substantially reduced. The uniformly low flare values observed in both groups—markedly below the ranges traditionally reported following standard phacoemulsification [23,24,25]—underscore the inherent low-trauma nature of Eight-chop and help explain why further lowering IOP conferred no measurable inflammatory advantage.

Corneal endothelial cell density loss further illustrates this concept. Endothelial preservation in this series was exceptional: losses remained near 1% at 7 and 19 weeks, far below typical values reported in the literature for conventional techniques [26,27]. Importantly, CECD loss did not differ significantly between IOP settings. This finding indicates that the protective factors governing endothelial safety during Eight-chop reside primarily in efficient fragmentation mechanics, reduced ultrasound usage, and controlled fluidic behavior rather than in moderate variations in IOP settings within the tested range. Linear mixed-effects analysis identified lens hardness as the only consistent predictor of endothelial loss, consistent with longstanding observations that nuclear density is a dominant determinant of endothelial stress [8,28]. The absence of an association between flare and CECD loss suggests that endothelial injury under Eight-chop is influenced more by direct mechanical factors than by postoperative inflammatory pathways, further emphasizing the technique’s structural advantages.

Corneal morphological parameters, CCT, CV, and PHC, demonstrated stable recovery patterns and showed no meaningful differences between the two IOP settings. These metrics provide important structural confirmation that the Eight-chop technique minimizes endothelial deformation and maintains corneal biomechanical homeostasis after surgery. The normalization of CV and PHC over time further supports the notion that this surgical approach imposes minimal morphological stress on the corneal endothelium.

Postoperative IOP reductions were similar between the two IOP groups and consistent with the well-documented IOP-lowering effect of cataract extraction [29,30]. Mixed-effects modeling showed no relationship between flare intensity and postoperative IOP reduction, indicating that early postoperative inflammation does not significantly influence trabecular outflow recovery when surgical trauma is minimized. This stability has clinical significance, particularly for patients with pre-existing glaucoma risk.

Taken together, the overall findings suggest that the principal determinants of minimally invasive cataract surgery in the present setting emerge from the Eight-chop technique itself rather than from modulation of intraoperative IOP within the conventional and low-pressure ranges tested. Notably, phaco time and aspiration time were significantly longer in the low-IOP group, indicating reduced procedural efficiency under lower pressure conditions. Prolonged ultrasound activation and aspiration may increase intraocular manipulation and cumulative fluidic exposure, which could partially offset any theoretical benefits of IOP reduction. In the present study, these efficiency-related trade-offs may have contributed to the absence of additional endothelial or inflammatory advantages in the low-IOP setting, despite the anticipated reduction in mechanical stress. These findings suggest that optimization of intraoperative IOP should consider not only pressure-related factors but also their impact on surgical efficiency and total intraocular stress. The technique’s mechanical segmentation strategy reduces the dependence on ultrasound power, stabilizes fluidics, and reliably preserves intraocular tissues. Under these conditions, lowering IOP provides no additional measurable advantage and may be associated with a modest reduction in surgical efficiency, potentially due to impaired fragment followability.

Importantly, the present study was not designed to compare the Eight-chop technique with other nucleus fragmentation methods, nor to establish its superiority over fluidic parameters or surgical strategies in general. Rather, the null findings observed under different intraoperative IOP settings should be interpreted strictly within the context of the predefined hypothesis—that modulation of IOP alone does not substantially alter short-term surgical invasiveness when chamber stability is already ensured by a low-invasive fragmentation strategy. Accordingly, the absence of additional benefit from lowering IOP should not be construed as proof of the inherent robustness or dominance of the Eight-chop technique itself, but rather as evidence that, under the present experimental conditions, further IOP reduction provides no incremental advantage beyond the technique’s baseline stability.

These results carry meaningful implications for clinical practice. Surgeons may confidently maintain conventional IOP settings when using Eight-chop under active fluidics without concern of imposing additional stress on ocular tissues. For patients with fragile corneal endothelium, such as those with borderline cell counts or early dystrophic changes, Eight-chop remains a compelling option due to its intrinsic structural advantages. In settings where anterior chamber depth is shallow or where nuclear density is high—circumstances that typically increase surgical trauma—Eight-chop may be particularly beneficial. Furthermore, the technique may represent an attractive alternative to femtosecond laser–assisted cataract surgery, especially in situations where cost, accessibility, or nuclear fragmentation efficacy is a limiting factor.

The fluidic behavior observed in this study can be understood in the context of the structural characteristics of the Eight-chop–based surgical strategy. Because the nucleus is fully divided into eight small fragments at the outset, each fragment moves efficiently toward the phaco tip, enabling rapid aspiration and reducing the amount of irrigation required. The technique does not require a side-port incision or the use of a second instrument, thereby eliminating paracentesis leakage and preventing globe deformation that can disturb outflow at the main incision. These features collectively enhance chamber stability and reduce mechanical stress on the corneal endothelium, helping to explain the consistently favorable recovery patterns observed across both IOP settings. The findings of this study also provide a broader perspective on the future trajectory of cataract surgery. Current trends diverge into two contrasting directions: increasingly complex phacoemulsification platforms incorporating technologies such as AFS and Active Sentry, and low-technology, non-ultrasound extraction approaches modeled after manual small-incision cataract surgery [31]. The present results suggest that, when an efficient fragmentation strategy such as Eight-chop is employed, the marginal benefit of additional chamber-stabilizing technologies may be limited, raising the possibility that device complexity and cost exceed what is necessary for routine surgery. Conversely, non-ultrasound strategies often struggle with extremely dense nuclei, particularly when traditional fragmentation methods such as divide-and-conquer or phaco-chop are used. Integrating Eight-chop with emerging low-cost extraction platforms—such as the miCOR system [32,33,34]—may offer a practical hybrid solution. Such an approach could maintain minimally invasive surgery through ≤3 mm incisions, reduce energy delivery and irrigation volume, and expand accessibility to high-quality cataract care across diverse clinical environments.

Despite its strengths, this study has several limitations. First, the findings reflect the performance of Eight-chop phacoemulsification under two specific IOP settings and cannot be directly generalized to other nuclear fragmentation techniques or surgical strategies. Because the mechanical behavior of lens fragments, fluidic flow patterns, and vacuum engagement differ substantially across techniques, the present results should be interpreted within the context of an Eight-chop-based surgical strategy. Second, this study was conducted at a single center by a single experienced surgeon using the Eight-chop technique, which represents a highly specialized fragmentation strategy. Although this design ensured strict procedural consistency and minimized inter-operator variability, it inevitably limits the generalizability of the findings. The observed lack of additional benefit from lowering intraoperative IOP under the present study conditions may not be directly applicable to other surgeons, institutions, or more commonly used nucleus fragmentation techniques such as phaco-chop or divide-and-conquer, in which fluidic demands and chamber stability characteristics differ substantially. Third, although intraoperative chamber stability was qualitatively assessed and no clinically significant surge or instability was observed, anterior chamber behavior was not quantitatively measured using objective metrics. Therefore, subtle differences in fluidic dynamics between pressure settings may have gone undetected. Fourth, the minimum IOP achievable in this study was constrained by the technical specifications of the OZil ultrasound handpiece. Pressures below 28 mmHg could not be evaluated, and it remains unknown whether further reductions—if technically feasible—would yield different fluidic or mechanical outcomes. Fifth, because the Active Sentry handpiece was not used, real-time surge mitigation was not assessed, and the present findings cannot be extrapolated to conditions involving active pressure-sensing technology. Future studies incorporating both low- and high-IOP settings with the Active Sentry handpiece will be necessary to determine whether automated surge control modifies the interaction between IOP and intraoperative performance. Sixth, although sex distribution was recorded, the present study was not powered to detect sex-specific differences in surgical outcomes; therefore, potential sex-related differences in endothelial response or fluidic behavior were not analyzed and warrant investigation in larger future studies. Seventh, surgical invasiveness in the present study was assessed using established surrogate markers, including CECD loss, aqueous flare, and operative parameters, rather than direct measurements of anterior chamber stability, surge events, or real-time intraoperative IOP fluctuations. Although these indicators are widely accepted proxies for intraocular trauma and postoperative inflammation, the absence of direct fluidic measurements and patient-centered outcomes such as visual recovery or subjective comfort represents a limitation of this study and should be considered when interpreting the results. Eighth, from a statistical perspective, no adjustment for multiple comparisons was applied in the present study because the analyses were hypothesis-driven and based on a paired-eye design, which is commonly used in cataract surgery research. Nevertheless, given the very small expected effect sizes and the low incidence of postoperative adverse events under the Eight-chop strategy, the possibility of type II error—particularly for secondary outcomes—cannot be excluded. In addition, postoperative increases in CECD were treated as 0% loss based on established test–retest variability of specular microscopy, and this approach was applied uniformly to both IOP groups to avoid overestimation of endothelial damage. Finally, the follow-up period was limited to 19 weeks, preventing assessment of long-term endothelial behavior under different IOP conditions. In addition, the present cohort predominantly consisted of eyes with mild to moderate nuclear density and anatomically stable anterior segments. Surgical behavior and the influence of intraoperative IOP modulation may differ in more challenging scenarios, such as dense nuclear cataracts (Emery-Little Grade > III), shallow anterior chambers, or glaucomatous eyes. Therefore, the applicability of the present findings to these higher-risk populations requires further investigation.

## 5. Conclusions

The findings of this fellow-eye investigation demonstrate that lowering intraoperative IOP from conventional levels to near-physiologic values does not confer additional surgical benefit when Eight-chop phacoemulsification is performed under active-fluidics control. The intrinsic mechanical and fluidic advantages of the Eight-chop technique—complete pre-segmentation of the nucleus, improved fragment followability, and the absence of side-port leakage or globe distortion—appear to exert a dominant influence on postoperative inflammatory and endothelial responses, resulting in consistently low surgical invasiveness irrespective of IOP setting. These results support the use of standard pressure settings during Eight-chop phacoemulsification and further underscore the technique’s robustness and adaptability across a wide range of surgical environments. At the same time, the present findings highlight the potential value of simplified, energy-efficient surgical strategies in the broader context of cataract surgery, where increasing system complexity and cost contrast with emerging low-technology alternatives. Future studies incorporating more challenging ocular conditions, alternative fluidics configurations, or next-generation extraction devices may help clarify whether specific subgroups could benefit from tailored IOP targets or hybrid low-energy approaches.

## Figures and Tables

**Table 1:** Preoperative characteristic.

Parameter	High IOP (n = 56)	Low IOP (n = 56)	*p*-Value *
Age (years)	73.4 ± 7.14	73.4 ± 7.14	N/A
Sex (M/F)	20/36	20/36	N/A
Axial length (mm)	24.1 ± 1.59	24.1 ± 1.64	0.943
Anterior chamber depth (mm)	3.21 ± 0.33	3.21 ± 0.35	0.664
Nucleus grade	2.27 ± 0.38	2.27 ± 0.36	0.844
CECD (cells/mm^2^)	2672 ± 235	2683 ± 228	0.449
CV (%)	38.9 ± 5.30	39.1 ± 5.75	0.663
PHC (%)	45.1 ± 7.50	45.4 ± 7.55	0.608
CCT (µm)	530 ± 38.0	530 ± 39.6	0.821
Preoperative IOP (mmHg)	13.4 ± 1.84	13.5 ± 1.60	0.623
Preoperative flare (ph/ms)	8.00 ± 2.63 Median 7.45 [6.4–8.8]	7.89 ± 2.22 Median 7.8 [6.0–9.2]	0.756

* *p*-values from linear mixed-effects models. N/A = Not applicable. Age and sex are identical between groups because each patient contributes one eye to each IOP setting (paired-eye design).

**Table 2:** Intraoperative surgical parameters.

Parameter	High IOP (n = 56)	Low IOP (n = 56)	*p*-Value *
Operative time (min)	4.82 ± 1.13	5.08 ± 1.10	0.082
Phaco time (s)	13.9 ± 4.40	16.2 ± 5.22	0.001
Aspiration time (s)	69.0 ± 17.9	75.0 ± 18.3	0.033
CDE	5.56 ± 1.9	5.93 ± 1.87	0.099
Irrigation volume (mL)	25.2 ± 7.35	26.6 ± 7.71	0.214

Linear mixed-effects model, fixed effect: IOP setting; random effect: patient ID. * *p*-values from linear mixed-effects models.

**Table 3:** Postoperative aqueous flare and endothelial cell density outcomes by IOP setting.

Parameter	High IOP	Low IOP	*p*-Value *
Flare (ph/ms)—Preoperative	8.00 ± 2.63 (n = 54)	7.89 ± 2.22 (n = 55)	0.756
Flare (ph/ms)—Day 1	14.54 ± 4.76 (n = 53)	14.84 ± 5.10 (n = 55)	0.655
Flare (ph/ms)—Week 1	17.54 ± 7.67 (n = 54)	17.80 ± 8.18 (n = 55)	0.820
Flare (ph/ms)—Week 7	12.64 ± 3.93 (n = 51)	12.44 ± 3.93 (n = 51)	0.700
Flare (ph/ms)—Week 19	10.68 ± 2.41 (n = 44)	10.54 ± 2.12 (n = 44)	0.749
CECD loss (%)—Week 7	−0.98 ± 1.16 (n = 49)	−0.82 ± 1.05 (n = 49)	0.460
CECD loss (%)—Week 19	−1.28 ± 1.69 (n = 41)	−0.93 ± 1.38 (n = 41)	0.239

Linear mixed-effects model structure: fixed effect = IOP setting; random effect = patient ID. * *p*-values from linear mixed-effects models.

**Table 4:** Comparison of corneal morphological parameters between high- and low-IOP settings.

Parameter	Time Point	High IOP (Mean ± SD)	Low IOP (Mean ± SD)	*p*-Value *
CCT (µm)	Preoperative	529.9 ± 38.0 (n = 56)	530.3 ± 39.6 (n = 56)	0.821
CCT (µm)	Week 7	529.9 ± 39.8 (n = 49)	528.2 ± 40.6 (n = 49)	0.410
CCT (µm)	Week 19	530.4 ± 37.1 (n = 41)	530.6 ± 38.4 (n = 41)	0.942
CV (%)	Preoperative	38.9 ± 5.3 (n = 56)	39.1 ± 5.7 (n = 56)	0.663
CV (%)	Week 7	38.7 ± 6.0 (n = 49)	38.0 ± 5.1 (n = 49)	0.190
CV (%)	Week 19	38.5 ± 4.9 (n = 41)	38.0 ± 4.5 (n = 41)	0.386
PHC (%)	Preoperative	45.1 ± 7.5 (n = 56)	45.4 ± 7.6 (n = 56)	0.608
PHC (%)	Week 7	45.9 ± 8.0 (n = 49)	46.5 ± 6.6 (n = 49)	0.389
PHC (%)	Week 19	46.0 ± 6.1 (n = 41)	46.6 ± 6.2 (n = 41)	0.459

* *p*-values calculated using a linear mixed-effects models.

**Table 5:** Changes in intraocular pressure.

Time Point	Setting	n	Mean ± SD (mmHg)	% Change	*p*-Value *
Preoperative IOP	High	56	13.37 ± 1.84	N/A	0.623
Preoperative IOP	Low	55	13.44 ± 1.60	N/A	
7 weeks postoperative	High	51	12.19 ± 1.69	−8.8%	0.836
7 weeks postoperative	Low	51	12.22 ± 1.55	−9.1%	
19 weeks postoperative	High	43	12.42 ± 1.97	−7.1%	0.528
19 weeks postoperative	Low	43	12.29 ± 1.87	−8.6%	

* *p*-values from linear mixed-effects models comparing high vs. low settings. N/A = Not applicable.

**Table 6:** Comparison of best-corrected visual acuity between high- and low-IOP Settings.

Time Point	High IOP (Mean ± SD)	Low IOP (Mean ± SD)	*p*-Value *
Preoperative	0.097 ± 0.214 (n = 56)	0.113 ± 0.131 (n = 56)	0.597
Week 7	−0.069 ± 0.029 (n = 51)	−0.071 ± 0.022 (n = 51)	0.644
Week 19	−0.072 ± 0.024 (n = 43)	−0.068 ± 0.028 (n = 43)	0.336

* *p*-values calculated using a linear mixed-effects models.

## Data Availability

The original contributions presented in this study are included in the article/Supplementary Material. Further inquiries can be directed to the corresponding author.

## Supplementary Materials

The following supporting information can be downloaded at https://www.mdpi.com/article/10.3390/jcm15010366/s1, Supplementary Dataset 1: Anonymized minimal dataset for the low-IOP analysis; Supplementary Dataset 2: Anonymized full dataset for the low-IOP analysis.

## Abbreviations

The following abbreviations are used in this manuscript:

AFS	Active Fluidics System
ASM	Active Surge Mitigation
CCT	Central corneal thickness
CDE	Cumulative dissipated energy
CECD	Corneal endothelial cell density
CV	Coefficient of variation
GFS	Gravity-based fluidics system
IOP	Intraocular pressure
PHC	Percentage of hexagonal cells
